# Rapidly Progressive Post-Infarction Left Ventricular Aneurysm: Multimodality Imaging-Guided Assessment of Adverse Remodeling and Surgical Ventricular Restoration

**DOI:** 10.3390/jcm15145516

**Published:** 2026-07-14

**Authors:** Alina Craciun-Mirescu, Oana Munteanu Mirea, Despina Emanuela Toader, Denisa Epingeac, Constantin Militaru, Victor Raicea

**Affiliations:** 1 Department of Cardiology, Emergency County Clinical Hospital, 200642 Craiova, Romania; oana.mirea83@gmail.com (O.M.M.); despinamtoader@yahoo.com (D.E.T.); cccmilitaru@yahoo.com (C.M.); 2 Faculty of Medicine, University of Medicine and Pharmacy of Craiova, 200349 Craiova, Romania; drraiceavictor@gmail.com; 3 Department of Cardiovascular Surgery, Emergency County Clinical Hospital, 200642 Craiova, Romania

**Keywords:** left ventricular aneurysm, adverse remodeling, ventricular geometry, multimodality imaging, surgical ventricular restoration, cardiovascular magnetic resonance

## Abstract

**Background:** Rapid, disproportionate expansion of post-infarction left ventricular (LV) aneurysms represents a high-risk remodeling phenotype characterized by progressive mechanical deterioration and severe geometric distortion. **Methods:** A 54-year-old male presented with late anterior myocardial infarction complicated by a partially thrombosed apical LV aneurysm with an initial left ventricular ejection fraction (LVEF) of 35%. Despite clinical stability under optimal guideline-directed medical therapy, serial multimodality imaging at 7 weeks revealed an aggressive, disproportionate expansion of the aneurysmal component to 120 mL, inducing severe ventricular geometric distortion and secondary functional degradation (LVEF 20%). Multimodality imaging demonstrated a favorable geometry of the functional ventricle with preserved contractile function. **Results:** The patient underwent prompt surgical ventricular restoration using a double-patch Dor technique, effectively excluding the large aneurysm and restoring physiological ventricular geometry. The postoperative course was uneventful. At 6-month follow-up, cardiovascular magnetic resonance confirmed sustained reverse remodeling and significant recovery of systolic LV function (LVEF 47%). **Conclusions:** This case illustrates that rapid post-infarction aneurysmal expansion may occur despite apparent clinical stability. Comprehensive multimodality imaging may help identify selected patients in whom a reconstructible myocardial substrate supports surgical ventricular restoration despite severely reduced LVEF, even when conventional clinical indications for aneurysmectomy are absent.

## 1. Introduction

Left ventricular (LV) aneurysm remains one of the most important mechanical complications of transmural ST-segment elevation myocardial infarction (STEMI), although its incidence has markedly declined following the widespread implementation of timely primary percutaneous coronary intervention. It occurs predominantly after large infarctions associated with delayed presentation or unsuccessful reperfusion and remains associated with adverse ventricular remodeling, progressive heart failure, ventricular arrhythmias, systemic thromboembolism, and increased long-term mortality [1,2]. In most patients, post-infarction LV aneurysm formation follows a gradual remodeling process over weeks to months, with progressive ventricular adaptation and eventual structural stabilization [2].

However, ventricular remodeling is not uniform, and substantial interindividual variability exists in both its rate and severity. A small subset of patients develops a high-risk remodeling phenotype characterized by rapid infarct expansion, disproportionate aneurysmal enlargement, progressive geometric distortion, marked wall thinning, and rapid deterioration of ventricular mechanics over a relatively short period [3]. The mechanisms underlying this aggressive phenotype remain incompletely understood but are thought to involve delayed reperfusion, extensive transmural myocardial necrosis, infarct expansion, adverse extracellular matrix remodeling, and persistent biomechanical stress [2,3]. Progressive aneurysm enlargement accompanied by severe wall thinning and geometric distortion may reflect advanced structural instability and an increased risk of life-threatening mechanical complications [4]. Although isolated cases of rapidly progressive post-infarction LV aneurysms have been reported, this pattern of evolution remains uncommon, and the mechanisms underlying this accelerated evolution, as well as the imaging characteristics that should prompt surgical intervention, are not well defined. Importantly, significant structural deterioration may occur despite apparent clinical stability, allowing advanced adverse remodeling to remain clinically unrecognized until severe ventricular dysfunction or major mechanical complications develop [3].

Although surgical ventricular restoration (SVR) remains an established therapeutic option for selected patients with ischemic LV aneurysms, optimal patient selection continues to be debated following the Surgical Treatment for Ischemic Heart Failure (STICH) trial and subsequent analyses [5,6]. Increasing evidence suggests that successful patient selection for SVR relies not only on global left ventricular ejection fraction (LVEF), but also on a comprehensive assessment of ventricular geometry, the size and configuration of the residual functional ventricular cavity, myocardial viability, and scar distribution [6,7]. Contemporary multimodality imaging, including transthoracic echocardiography, three-dimensional echocardiography, cardiovascular magnetic resonance (CMR), and cardiac computed tomography (CT), provides complementary structural and functional information beyond conventional assessment of global systolic function [8,9,10]. Serial imaging enables accurate evaluation of ventricular geometry, remodeling dynamics, tissue characterization, thrombus detection, and mechanical stability, thereby refining individualized surgical decision-making in selected patients [9,11].

The present report describes an unusual case of rapidly progressive post-infarction LV aneurysm demonstrating marked geometric deterioration over only seven weeks despite guideline-directed medical therapy and apparent clinical stability. Serial multimodality imaging documented disproportionate aneurysmal expansion while simultaneously demonstrating preservation of a reconstructible myocardial substrate suitable for surgical reconstruction. Although the patient did not meet conventional indications for aneurysmectomy, the rapid structural progression together with imaging findings suggestive of increasing mechanical instability raised concern for an increased risk of life-threatening mechanical complications and prompted early surgical ventricular restoration.

This case highlights the potential value of comprehensive serial multimodality imaging for identifying accelerated adverse remodeling, refining individualized surgical decision-making, and recognizing patients who may benefit from early intervention before irreversible ventricular deterioration or life-threatening mechanical complications occur. As a single-patient observation, these findings should be considered hypothesis-generating and interpreted within the context of current evidence rather than as a basis for modifying existing therapeutic recommendations.

## 2. Case Presentation

### 2.1. Clinical Presentation and Initial Assessment

A 54-year-old male active smoker with no previous cardiovascular disease, with smoking as his only known cardiovascular risk factor, and no regular medical treatment, presented with progressive exertional dyspnea following an episode of severe chest pain approximately 14 days prior to admission. On presentation, he was hemodynamically stable (blood pressure 110/65 mmHg, heart rate 111 beats/min), with an oxygen saturation of 97% on room air. Physical examination revealed Killip class I heart failure (NYHA functional class II), with no pulmonary crackles or peripheral edema. Cardiac auscultation demonstrated a regular rhythm without pathological murmurs. The admission electrocardiogram showed sinus rhythm with persistent ST-segment elevation in the anterior precordial leads, pathological Q waves, and T-wave inversion in the inferior and anterolateral leads, consistent with a late-presenting anterior ST-segment elevation myocardial infarction. Cardiac biomarkers were elevated, confirming myocardial necrosis. Initial transthoracic echocardiography demonstrated mild LV dilatation predominantly related to a partially thrombosed apical aneurysm with an estimated volume of approximately 25 mL, while the remaining contractile left ventricle was relatively preserved in size and contractile performance, maintaining an overall baseline LVEF of approximately 35% (Figure 1A). Coronary angiography revealed recent occlusion of the mid-distal left anterior descending artery; revascularization was deferred based on unfavorable coronary anatomy and the absence of viable myocardium within the target territory (Figure 1B). Cardiac computed tomography confirmed a true apical aneurysm with preserved myocardial continuity, excluding a pseudoaneurysm (Figure 1C). During a 17-day hospitalization, the patient had a favorable clinical course under guideline-directed medical therapy. Because of the documented LV apical thrombus, the patient received triple antithrombotic therapy with aspirin, clopidogrel, and rivaroxaban during the first two weeks of hospitalization. Clopidogrel was subsequently discontinued, and the patient was discharged receiving aspirin 75 mg once daily, rivaroxaban 20 mg once daily, atorvastatin 80 mg once daily, dapagliflozin 10 mg once daily, perindopril 5 mg once daily, metoprolol succinate 100 mg once daily, ivabradine 5 mg twice daily, furosemide 20 mg once daily, and pantoprazole 40 mg once daily.

### 2.2. Multimodality Imaging and Remodeling Progression

Cardiac magnetic resonance imaging performed 3 weeks after the initial presentation provided complementary volumetric and tissue characterization, demonstrating global LV dilatation (LV end-diastolic volume 190 mL [LVEDVi 111 mL/m^2^], LV end-systolic volume 128 mL [LVESVi 74 mL/m^2^]) with severely reduced systolic function (LVEF 33%). Sequential analysis highlighted a rapid doubling of the aneurysmal component to approximately 50 mL, whereas the remaining contractile left ventricle measured approximately 140 mL with preserved contractility. Late gadolinium enhancement identified transmural myocardial infarction of the apical and anterior walls, together with a low-signal apical thrombus (Figure 2A; see Supplementary Video S1) [4].

Despite apparent clinical stability under guideline-directed medical therapy, follow-up transthoracic echocardiography performed seven weeks after presentation demonstrated rapid and disproportionate expansion of the aneurysmal sac, to a volume of 120 mL. This rapid expansion resulted in volumetric dominance of the aneurysmal segment over the functional ventricle, causing marked geometric distortion and severe global functional deterioration (LVEF 20%). Imaging findings suggested advanced adverse remodeling characterized by severe wall thinning, increased wall stress, and progressive structural instability. Nevertheless, the remaining functional LV preserved favorable geometry and contractile characteristics, indicating preservation of a myocardial substrate suitable for surgical ventricular restoration (Figure 2B,C; see Supplementary Video S2).

### 2.3. Imaging-Guided Therapeutic Decision

Given the rapid geometric distortion and the progressive LV functional deterioration, the case was discussed by the multidisciplinary Heart Team. Although conventional clinical indications for aneurysmectomy—such as refractory NYHA class III-IV heart failure, angina pectoris, or malignant ventricular arrhythmias—were absent, serial multimodality imaging demonstrated progressive structural instability and LV functional deterioration, together with preservation of a reconstructible ventricle. The coexistence of these imaging findings prompted early surgical ventricular restoration before irreversible deterioration of ventricular geometry and function, or life-threatening mechanical complications occurred [5,6].

### 2.4. Surgical Ventricular Restoration

The patient underwent prompt surgical ventricular restoration with exclusion of the noncontractile aneurysmal segment and evacuation of the intraventricular thrombus. Reconstruction was performed using an endoventricular double-patch Dor technique aimed at restoring a physiological elliptical ventricular shape while preserving an adequate functional ventricular volume (Figure 3A) [12,13].

The procedure was performed under conventional cardiopulmonary bypass with moderate systemic hypothermia (28 °C). Intraoperatively, dense pericardial adhesions over the infarcted apex and a large, laminated thrombus substantially increased operative complexity, requiring careful myocardial handling to minimize the risk of systemic thromboembolism (Figure 3B). Separation from cardiopulmonary bypass was achieved smoothly without mechanical circulatory support.

### 2.5. Clinical and Imaging Follow-Up

Postoperative recovery was uneventful. The patient required a 5-day intensive care unit stay and was discharged in stable clinical condition on postoperative day 10. Early postoperative echocardiography demonstrated an improvement in LVEF to 45%. Following surgical ventricular restoration and complete thrombus evacuation, antithrombotic therapy was modified by replacing rivaroxaban with a vitamin K antagonist (acenocumarol), while aspirin and guideline-directed heart failure therapy were continued.

At 6-month follow-up, CMR imaging confirmed significant and sustained reverse ventricular remodeling with marked reduction in global ventricular volumes (LVEDV decreased from 192 to 145 mL; LVESV decreased from 128 to 77 mL) and sustained recovery of global systolic function (LVEF 47%) (Figure 4; see Supplementary Video S3).

## 3. Discussion

Most post-infarction left ventricular aneurysms evolve gradually over weeks to months before reaching relative structural stabilization [2,3]. During this period, patients may develop symptoms related to heart failure, ventricular arrhythmias, persistent angina, or thromboembolic complications. Consequently, therapeutic decision-making has traditionally relied on clinical parameters together with the severity of global systolic dysfunction [1].

Current management of post-infarction LV aneurysms is guided by clinical presentation, ventricular function, and aneurysm-related complications [1]. Guideline-directed medical therapy for heart failure remains the cornerstone of treatment, while anticoagulation is generally recommended in the presence of LV thrombus or high thromboembolic risk [1,14]. Surgical ventricular restoration is generally reserved for carefully selected patients with refractory heart failure, ventricular arrhythmias, recurrent thromboembolism, or large dyskinetic aneurysms in whom restoration of ventricular geometry is expected to improve functional outcome [5,6].

In contrast, the present case followed a different pattern of remodeling-a rapidly progressive post-infarction left ventricular aneurysm evolving over a remarkably brief time frame despite optimal medical therapy and apparent clinical stability. This rapid progression underscores the distinct pathophysiological heterogeneity of ventricular remodeling following transmural infarction [3]. In terms of myocardial mechanics, this accelerated expansion was most likely related to the extensive transmural infarction and persistent biomechanical stress acting on the infarct border zone [2,4]. No additional conditions known to promote accelerated aneurysm progression, such as connective tissue disorders or inflammatory diseases, were identified. The absence of significant clinical deterioration despite severe adverse remodeling highlights the discrepancy between apparent clinical stability and underlying progressive structural deterioration, confirming that adverse ventricular remodeling may progress silently until severe global functional impairment develops [3].

Although rapidly progressive post-infarction LV aneurysms have been only rarely described, this case illustrates that adverse remodeling may occasionally evolve far more rapidly and silently than expected, emphasizing the importance of multimodality imaging for identifying accelerated remodeling and facilitating timely surgical evaluation before conventional clinical indications become evident.

Left ventricular geometry represents a key determinant of global cardiac performance and clinical prognosis following myocardial infarction [4,7]. In the present case, the rapid volumetric dominance of the noncontractile aneurysmal sac over the functional myocardium led to severe mechanical disruption with geometric distortion emerging as the principal driver of global functional impairment rather than intrinsic myocardial contractility. This observation reinforces the concept that ventricular geometry may outweigh conventional global systolic indices when assessing suitability for ventricular reconstruction in selected patients [4,7].

Importantly, advanced multimodality imaging with cardiovascular magnetic resonance and three-dimensional echocardiography provided the structural basis for individualized therapeutic decision-making. Contemporary recommendations recognize transthoracic echocardiography as the first-line imaging modality for evaluating LV aneurysm morphology and ventricular function, whereas cardiovascular magnetic resonance represents the reference standard for ventricular volumes, myocardial viability, tissue characterization, and thrombus assessment. Cardiac computed tomography provides complementary anatomical information, particularly for differentiating true aneurysms from pseudoaneurysms and facilitating preoperative planning [8,9,10]. Despite severely reduced global LVEF, imaging demonstrated preserved contractility and a favorable geometry within the remaining functional LV cavity. The clear delineation between viable and non-viable myocardium defined the optimal plane for surgical ventricular reconstruction [9]. These findings suggested that global systolic dysfunction was predominantly driven by geometric distortion and volumetric predominance of the aneurysmal component rather than irreversible failure of the residual functional ventricle, thereby supporting the feasibility of ventricular restoration.

Surgical exclusion of the noncontractile aneurysmal segment aims to reduce the total LV end-systolic volume index (LVESVI), restore a more physiological elliptical ventricular geometry, and reduce global wall stress, thereby optimizing the mechanical performance of the reconstructed left ventricle [4,12]. Large surgical registries have established that postoperative survival and functional recovery are strongly determined by the ability to achieve a postoperative LVESV < 70 mL/m^2^, alongside the extent of baseline myocardial viability [7,13]. The substantial reduction in LV volumes and sustained recovery of systolic function (LVEF 47%) documented by CMR at 6 months in this patient support this geometry-based surgical approach.

The optimal timing and specific role of surgical ventricular restoration remain subjects of ongoing debate, particularly following the long-term findings of the STICH (Surgical Treatment for Ischemic Heart Failure) trial [5,6]. While the STICH hypothesis did not demonstrate a significant survival advantage for routine surgical ventricular reconstruction combined with CABG versus CABG alone in unselected cohorts, subsequent post hoc analyses and contemporary surgical series have emphasized that careful patient selection based on ventricular geometry and postoperative volumetric targets is critical [6].

In this patient, the surgical indication was driven by imaging evidence suggesting structural instability and accelerated adverse remodeling rather than conventional clinical triggers, such as refractory heart failure or ventricular tachyarrhythmias [5,6]. While a severely depressed global LVEF (20%) is traditionally viewed as a marker of high operative risk and advanced ventricular dysfunction, our imaging-guided approach identified a favorable reconstructible ventricular geometry [6,7]. This observation highlights the importance of individualized, imaging-guided therapeutic decision-making based on ventricular geometry and function, remodeling dynamics and conventional clinical assessment rather than global systolic indices alone [3,6,7]. Future prospective studies are needed to determine whether serial multimodality imaging can further refine patient selection and optimize the timing of surgical ventricular restoration in patients with rapidly progressive post-infarction LV remodeling. Such an approach could facilitate earlier referral for surgical evaluation before irreversible ventricular deterioration limits the potential benefit of ventricular reconstruction.

## 4. Conclusions

Rapid, disproportionate expansion of a post-infarction left ventricular aneurysm represents an uncommon remodeling phenotype characterized by accelerated geometric deterioration and progressive impairment of ventricular mechanics. This case underscores the importance of serial multimodality imaging in identifying high-risk structural remodeling beyond symptom burden alone, particularly through assessment of ventricular geometry, remodeling dynamics, and residual reconstructive potential. Importantly, severely reduced global systolic function may not necessarily preclude ventricular reconstruction when a favorable residual ventricular geometry and preserved contractile substrate remain present. In selected patients, therapeutic decision-making may therefore benefit from integrating objective imaging markers of adverse remodeling together with assessment of residual ventricular geometry rather than relying exclusively on symptoms or global LVEF. Although this report represents an illustrative single-case observation and does not support modification of current therapeutic recommendations, it highlights the heterogenous remodeling course and the potential value of serial multimodality imaging in refining individualized surgical decision-making.

## Figures and Tables

**Figure 1:**
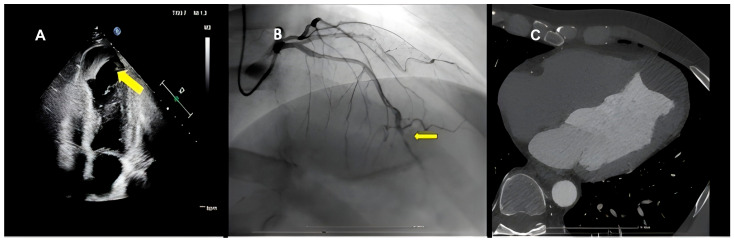
Initial multimodality imaging assessment at presentation. (**A**) Transthoracic echocardiography (apical four-chamber view) demonstrating mild left ventricular dilatation and a moderately sized, partially thrombosed apical aneurysm (yellow arrow). (**B**) Coronary angiography revealing chronic total occlusion of the mid-to-distal left anterior descending artery (yellow arrow). (**C**) Cardiac computed tomography angiography confirming true aneurysm morphology with preserved myocardial continuity and excluding pseudoaneurysm.

**Figure 2:**
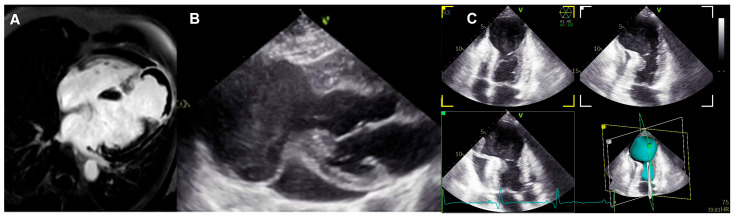
Serial multimodality imaging demonstrating rapid adverse remodeling and geometric distortion at follow-up. (**A**) Preoperative cardiovascular magnetic resonance imaging (long-axis view) showing extensive transmural apical infarction with wall thinning and early aneurysmal expansion containing a low-signal apical thrombus. (**B**) Two-dimensional parasternal long-axis echocardiography performed at 7 weeks demonstrating severe ventricular dilatation and aneurysmal progression. (**C**) Complementary three-dimensional volumetric echocardiographic analysis confirming advanced adverse ventricular remodeling with marked geometric distortion, volumetric predominance of the aneurysmal cavity over the residual functional ventricle, critically reduced ejection fraction, and preservation of a residual ventricular cavity suitable for reconstruction.

**Figure 3:**
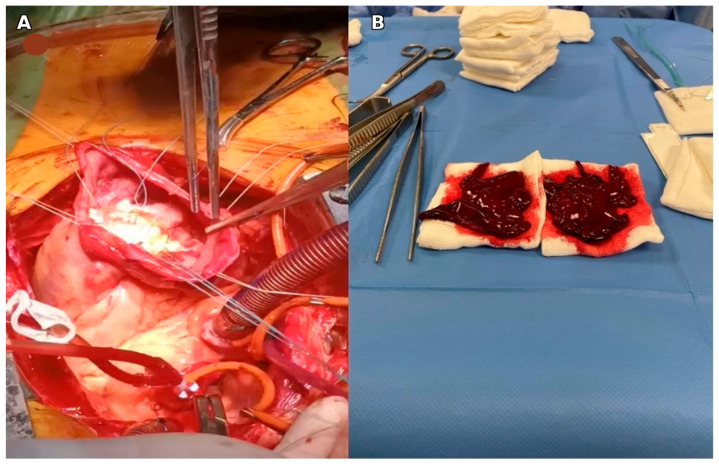
Intraoperative findings. (**A**) Resection of the aneurysmal segment and placement of an internal synthetic double-patch Dor reconstruction. (**B**) Large laminated intraventricular thrombus evacuated from the aneurysmal cavity.

**Figure 4:**
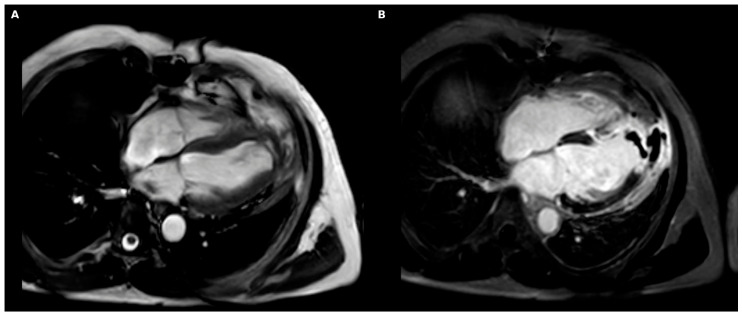
Six-month follow-up cardiovascular magnetic resonance imaging demonstrating postoperative reverse remodeling, reduction in left ventricular volumes, restoration of physiological ventricular geometry, and improvement in global systolic function (LVEF 47%). (**A**) Cine four-chamber view showing restoration of physiological left ventricular geometry with reduced ventricular volumes. (**B**) Late gadolinium enhancement image demonstrating postoperative exclusion of the aneurysmal sac and the reconstructed left ventricular cavity.

## Data Availability

The data supporting the findings of this study are available within the article and its Supplementary Materials. Additional data are not publicly available due to patient privacy and ethical restrictions.

## Supplementary Materials

The following supporting information can be downloaded at: https://www.mdpi.com/article/10.3390/jcm15145516/s1, Video S1: Preoperative cardiac magnetic resonance cine loop demonstrating rapid expansion of the apical left ventricular aneurysm; Video S2: Preoperative transthoracic echocardiography with three-dimensional volumetric analysis showing volumetric predominance of the aneurysmal cavity over the residual functional ventricle; Video S3: Six-month postoperative cardiac magnetic resonance cine loop demonstrating reverse remodeling, exclusion of the aneurysmal sac, restoration of physiological ventricular geometry, and recovery of global systolic function.

## Abbreviations

The following abbreviations are used in this manuscript:

CABG	Coronary artery bypass grafting
CMR	Cardiovascular magnetic resonance
CT	Computed tomography
ESC	European Society of Cardiology
LGE	Late gadolinium enhancement
LV	Left ventricle/left ventricular
LVEDV	Left ventricular end-diastolic volume
LVEDVi	Left ventricular end-diastolic volume index
LVEF	Left ventricular ejection fraction
LVESV	Left ventricular end-systolic volume
LVESVi	Left ventricular end-systolic volume index
NYHA	New York Heart Association
STEMI	ST-segment elevation myocardial infarction
